# Sequential methods for random-effects meta-analysis

**DOI:** 10.1002/sim.4088

**Published:** 2010-12-28

**Authors:** Julian P T Higgins, Anne Whitehead, Mark Simmonds

**Affiliations:** a MRC Biostatistics Unit, Institute of Public HealthRobinson Way, Cambridge CB2 0SR, U.K.; b Medical and Pharmaceutical Statistics Research Unit, Department of Mathematics and Statistics, Lancaster UniversityLancaster LA1 4YF, U.K.; c Wolfson Institute of Preventive Medicine, Bart's and The London School of Medicine and Dentistry, Queen Mary University of LondonLondon EC1M 6BQ, U.K.

**Keywords:** meta-analysis, sequential methods, cumulative meta-analysis, prospective meta-analysis, prior distributions

## Abstract

Although meta-analyses are typically viewed as retrospective activities, they are increasingly being applied prospectively to provide up-to-date evidence on specific research questions. When meta-analyses are updated account should be taken of the possibility of false-positive findings due to repeated significance tests. We discuss the use of sequential methods for meta-analyses that incorporate random effects to allow for heterogeneity across studies. We propose a method that uses an approximate semi-Bayes procedure to update evidence on the among-study variance, starting with an informative prior distribution that might be based on findings from previous meta-analyses. We compare our methods with other approaches, including the traditional method of cumulative meta-analysis, in a simulation study and observe that it has Type I and Type II error rates close to the nominal level. We illustrate the method using an example in the treatment of bleeding peptic ulcers. Copyright © 2010 John Wiley & Sons, Ltd.

## 1. Introduction

The meta-analysis of results from multiple, similar studies is traditionally considered a retrospective activity. However, many meta-analyses are updated over time as new studies are undertaken or are identified from the previously hidden literature. In particular, systematic reviews in the *Cochrane Database of Systematic Reviews* 1 are expected to be updated approximately every two years 2. Furthermore, genuinely prospective meta-analyses are increasingly being performed. For example, meta-analyses have been planned to combine evidence from existing, ongoing clinical trials 3, 4, and clinical trials have been designed with the aim of meta-analysing their results 5. In the field of genetic epidemiology, potentially important genetic variants are examined prospectively in multiple existing collections of DNA samples until sufficient evidence of their role has been determined 6, 7.

If a meta-analysis is conducted repeatedly on the addition of new studies, without any allowance for multiple testing, the overall risk of a false-positive finding will increase with the number of meta-analyses performed. Indeed, Berkey *et al*. note that when there is no underlying effect the process of continually updating a meta-analysis using standard significance tests will lead eventually to a false-positive result 8. One way to address this problem is to exploit formal sequential methods. Although sequential methods are well established in the analysis of individual randomized trials, they have received much less attention in the meta-analysis context. This is an area where opinions differ. Some argue that, because meta-analysts do not (typically) have control over the generation of new evidence, they are not in a position to act on stopping rules and so sequential methods should not be applied. In contrast, our view is that since meta-analysts frequently make decisions on whether to recommend further research or whether to recommend that there is convincing evidence for the presence or absence of an effect, sequential methods can be similarly important to meta-analyses as to individual primary studies. The intention is to facilitate a justifiable recommendation rather than to control future research directly.

Whitehead 9 describes the use of a stopping boundaries approach for fixed and random-effects meta-analyses based on the sequential trial methodology of Whitehead 10. Pogue and Yusuf suggest the use of monitoring boundaries based on alpha-spending functions and stochastic curtailment, with application to fixed-effect meta-analysis 11, and these have been implemented in practice 12. These methods are based on desirable sample sizes in terms of numbers of participants across all studies. Lan *et al.* apply the law of the iterated logarithm that ‘penalizes’ the usual test statistic to account for multiple tests, equivalent to using a particular open ended boundary in the framework of Whitehead, although the approach does not control Type II error 13, 14.

Heterogeneity of effects is a key characteristic of many meta-analyses, and estimation of the among-study variance may create problems in the early stages of a sequential meta-analysis. The methods of Pogue have been adapted to account for heterogeneity in a (retrospective) cumulative meta-analysis scenario, by adjusting the desired sample size based on a function of *I*^2^, a measure of inconsistency across the observed studies' findings 15, 16. The adjustment is derived essentially from a comparison of the (assumed known) variances of fixed-effect and random-effects meta-analysis estimates. In prospective meta-analyses these will not be known, although anticipated values of *I*^2^ might be used. Much of the present paper is devoted to the problem of accounting prospectively for heterogeneity, as it is the principal obstacle to the simple application of sequential trial methodology.

In this paper we consider the use of frequentist sequential approaches to meta-analysis with the aim of controlling the risk of drawing incorrect conclusions. We take an approach based on ‘group-sequential’ trial methodology, with a stopping rule. A previously proposed method is reviewed and extended to better incorporate random effects. We compare six methods (including a sequential fixed-effect method) through a simulation study and compare them in an example in the treatment of bleeding peptic ulcers. Our emphasis is on the determination of a straightforward approach that has good empirical properties, recognizing that even more formal methods are available through the use of dedicated software such as PEST 17.

## 2. A sequential approach to meta-analysis

### 2.1. Random-effects meta-analysis

Before introducing the sequential methods we review a general parametric approach to meta-analysis on which the sequential methods are based 18. Suppose we have an estimate, *y*_*i*_, of a true effect θ_*i*_ with estimated variance *v*_*i*_, from study *i*, *i* = 1, …, *k*. The effect might be measured as a log odds ratio or a difference in means for a comparative study, or a simple mean or logit proportion for a single group study; numerous other options are possible. We make the usual assumption that the estimates and variances are uncorrelated, although this will not always be the case (for example, if larger studies tend to be done in populations in which a treatment is less effective). Alternative approaches are available that do not rely on this assumption. For example, the raw data from the studies could be modelled, which may require individual participant data. Alternatively, all studies could be given equal weight 19, although recent proposals to adopt this practice have not been enthusiastically received 20–22.

A random-effects analysis involves inference on the distribution of the θ_*i*_ across studies. A standard assumption is that this distribution is normal with mean µ and variance τ^2^. The variance describes the extent of heterogeneity. A simple estimate of µ is given by the weighted average 
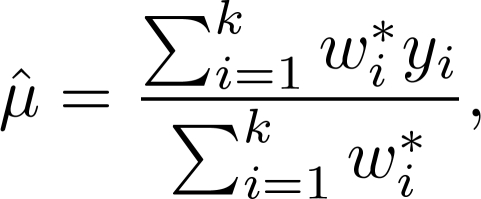
 where 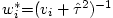 represents the weight attributed to study *i*. An approximate variance of this estimate is given by 
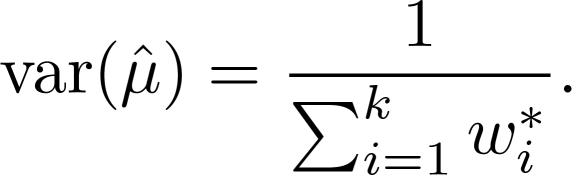
 The heterogeneity variance τ^2^ may be estimated simply by 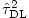, a method of moments estimator described by DerSimonian and Laird 23, where 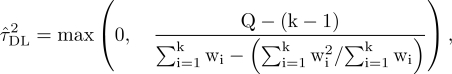 *w*_*i*_ = 1/*v*_*i*_and 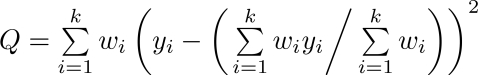 is the standard statistic used to test for heterogeneity across studies. In many circumstances, inferences from a random-effects meta-analysis should be based on the full distribution of the θ_*i*_ across studies rather than on µ alone. This can be achieved using a predictive interval for the effect in a new study, which takes into account both the mean µ and variance τ^2^ of effects 24. In the methods we describe below, we assume that primary inference is to be made on the mean effect across studies, i.e. on µ.

If 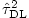 is equal to zero, then 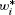 is equal to *w*_*i*_ and the resulting calculations lead to a fixed-effect meta-analysis.

The estimates *y*_*i*_ and *v*_*i*_ are typically calculated from either Wald statistics or score statistics. For the former, 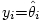 and 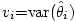, where 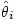 denotes the maximum likelihood estimate of θ_*i*_ and 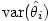 its variance. For the latter, we may write *y*_*i*_ = *Z*_*i*_/*V*_*i*_and *v*_*i*_ = 1/*V*_*i*_, where *Z*_*i*_ is the efficient score for θ_*i*_ and *V*_*i*_ is Fisher's information, both evaluated at θ_*i*_ = 0. When sample sizes are large and θ_*i*_ is reasonably near 0, there will be little difference between the results from the two approaches.

### 2.2. A monitoring boundary approach to sequential random-effects meta-analysis

To follow the progress of a meta-analysis over time we require a measure of the amount of relevant information contained within it. One approach is to use the total number of study participants 11, 15, 16. Pogue *et al.* introduce a desirable total that they refer to as the ‘optimal information size’, based on standard sample size calculations 11. Wetterslev *et al.* adapt this to account for observed heterogeneity in a retrospective cumulative meta-analysis 16, although in our applications this heterogeneity is yet to be observed. In contrast, Whitehead 9 and Lan *et al.* 13, 14 use statistical information in the form of the inverse variance of 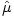 or, equivalently, the sum of the weights (specifically, Whitehead uses Fisher's information). We choose the statistical information approach, since it relates directly to the precision of the meta-analysis estimate and hence the amount of evidence contained in the collection of studies. In particular, if highly heterogeneous studies are combined sequentially over time, the precision of a random-effects meta-analytic estimate may decrease while the number of participants increases. Our approach may be used when study estimates *y*_*i*_ and *w*_*i*_ are calculated either from Wald statistics (as in Lan *et al.*) or score statistics (as in Whitehead).

A boundaries approach to sequential meta-analysis involves monitoring information using the sum of weights, 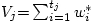 after *t*_*j*_ studies have been included, *j* = 1, 2, …. A plot of *V*_*j*_ against the cumulative effect size, 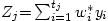, at each update of the meta-analysis provides a visualization of the path of the meta-analysis over time. A line from the origin to a point (*Z*_*j*_, *V*_*j*_) has slope equal to the simple random-effects meta-analytic estimate at the corresponding stage. Note that as information about τ^2^ changes over time, the individual study weights, 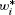, are recalculated on each update.

In the absence of heterogeneity, and when τ^2^ is assumed to be zero (as in a fixed-effect meta-analysis), this approach may be justified formally 9. It is equivalent to the group-sequential approach to monitoring clinical trials described by Whitehead 10. Monitoring boundaries may be used to stop the meta-analysis when there is sufficient evidence of an effect or lack of an effect based on a pre-specified significance level, power and clinically important effect.

**Figure 1:**
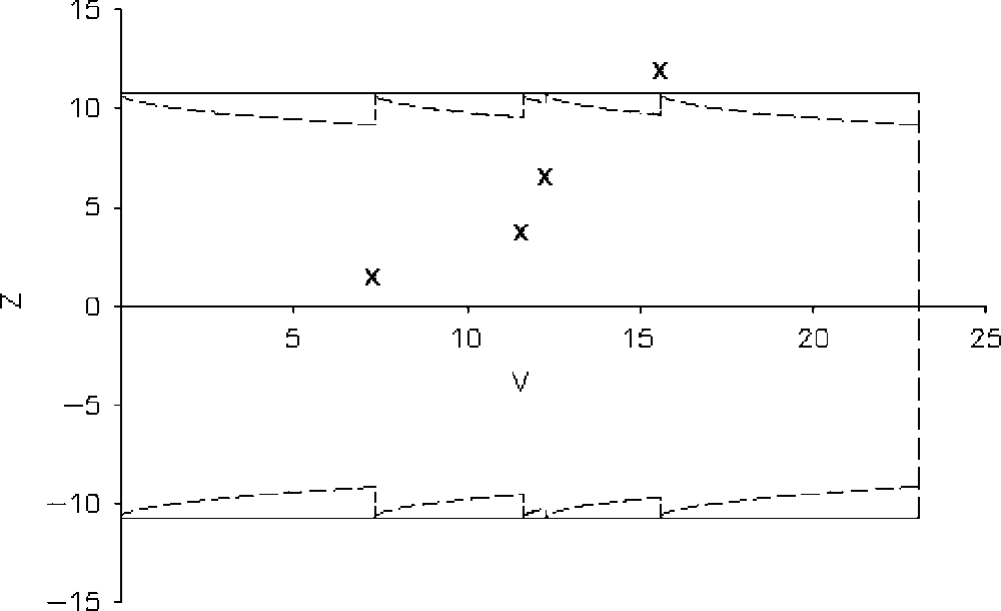
Sequential fixed-effect meta-analysis of peptic ulcers data.

Whitehead 9 describes the implementation of a triangular design in the context of a prospectively planned meta-analysis, and Bollen *et al.* implement a double-triangular design 25. Here we focus on the restricted procedure of Whitehead, equivalent to an O'Brien and Fleming stopping rule 26. The O'Brien and Fleming design produces a rectangular stopping boundary in the (*Z, V*) plane, with symmetric upper and lower boundaries at *Z* = ± *H* and a vertical boundary at *V* = *V*_max_ reflecting the maximum amount of information to be collected (Figure 1). Table I provides monitoring boundaries for a selection of choices of two-sided significance level (α) and power (1 − β), obtained using the software PEST 4.0 17. To allow for the possibility of the path crossing the boundary between updates, a ‘Christmas tree correction’ is applied, bringing the vertical boundaries in at update *j* to values 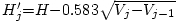 and 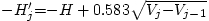, where *V*_0_ = 0 10.

**Table I:** Monitoring boundaries in the (*Z, V*) plane for the O'Brien and Fleming design.

		α		
		
1 − β	Boundary	0.001	0.01	0.05
0.8	Horizontal (±*H*)	±14.576	±9.779	±6.457
	Vertical (*V*_max_)	17.535	12.138	8.299
0.9	Horizontal (±*H*)	±16.120	±11.029	±7.461
	Vertical (*V*_max_)	21.447	15.438	11.079
0.95	Horizontal (±*H*)	±17.394	±12.061	±8.288
	Vertical (*V*_max_)	24.972	18.461	13.673

Values presented correspond to a reference effect size of µ_*R*_ = 1. Boundaries for other values of µ_*R*_ are obtained by dividing *H* by µ_*R*_, and *V*_max_ by 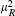.

As soon as |*Z*_*j*_|≥*H*′_*j*_ or *V*_*j*_≥*V*_max_, the meta-analysis will be stopped with the conclusion that the mean effect is in one direction or the other (for the former criterion) or that the mean effect is zero (if only the latter criterion is true). We refer to the values of *Z*_*j*_ and *V*_*j*_ at this point as *Z*_end_ and *V*_end_. If the path stops with *V*_end_>*V*_max_, then we extend the Christmas tree correction to *V*_end_, and consider an effect to be present if |*Z*_end_|≥*H*′_end_.

The methods we describe can be extended to address incremental information within studies as well as across studies. For instance, if at time *j*, study *i* has interim data yielding an estimate *y*_*ij*_ and weight 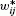 (based on the current estimate of τ^2^ across currently available data), then we can calculate 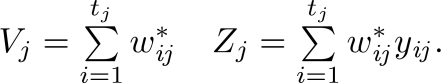 This situation was addressed by Whitehead 9 but we do not pursue it further in the present paper.

### 2.3. Repeated confidence intervals

The sequential procedure, described in the previous section in terms of monitoring boundaries, may also be presented using the repeated confidence intervals approach introduced by Jennison and Turnbull 27. This enables us to present sequential meta-analyses using forest plots, the conventional way of illustrating meta-analyses. Lower and upper limits, *L*_*j*_ and *U*_*j*_, for repeated confidence intervals after the inclusion of *t*_*j*_ studies can be obtained by inverting the sequential design, yielding 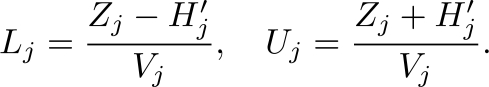
(1)The stopping rules based on the horizontal monitoring boundaries, that is stop the meta-analysis if *Z*_*j*_≤ − *H*′_*j*_ or if *Z*_*j*_≥*H*′_*j*_, is equivalent to stopping if the interval (*L*_*j*_, *U*_*j*_) excludes 0.

The repeated confidence intervals (*L*_*j*_, *U*_*j*_) have the property that all of them, calculated up until *V* = *V*_max_, contain the true value of the parameter µ with probability 1 − α, where αis the chosen two-sided significance level. When the amount of information is less than *V*_max_, the confidence interval series has probability greater than 1 − α that the truth is contained in every interval. This means that at any time, an individual confidence interval has probability greater than 1 − α of containing the truth, and is therefore wider than a confidence interval from a conventional meta-analysis.

The repeated confidence interval calculated at *V* = *V*_max_ is the last valid member of the sequence. Thus if the amount of information exceeds *V*_max_ it is unclear how to proceed. For consistency with the previous section, we propose the calculation of repeated confidence intervals until and including the first time that *V*_max_ is exceeded. Thereafter, this last confidence interval is used irrespective of how many additional data accumulate. This is likely to be conservative, but is based on the result that the coverage property of the valid sequence of repeated confidence intervals is also satisfied by the intersection of all of the repeated confidence intervals.

### 2.4. Formal versus ad hoc stopping rules

At each analysis the repeated confidence interval provides confidence limits that are valid and not dependent on any stopping rule which might be used. Thus information about the magnitude of µ can be obtained from the latest confidence interval. If the sequential meta-analysis follows a formal stopping rule, then a final analysis would be conducted after a stopping boundary had been crossed. A point estimate of µ is given by *Z*_end_/*V*_end_and the repeated confidence interval can be calculated. However, this point estimate may be a biased estimate. Methods for obtaining adjusted estimates and confidence intervals are available based on sequential methodology (see, for example, Chapter 5 of Whitehead 10), but these require specialized software, and we do not address them in detail here. The adjusted confidence interval will be narrower than that based on repeated confidence interval methodology. This is because the latter is not dependent on the stopping rule that has been used.

### 2.5. Estimation of heterogeneity variance

A key issue in sequential random-effects meta-analysis is the heterogeneity variance parameter, τ^2^. In the early stages of a sequential procedure, when very few studies are included, uncertainty surrounding any estimate of among-study variance will be considerable. Lan *et al.* prefer to over-estimate τ^2^ by using the arithmetic mean of the *y*_*i*_ when there are five or fewer studies, mainly to avoid spurious estimates of zero 13, 14. Whichever estimate is used, consecutive point estimates of the parameter are likely to be variable, and if 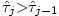 it is possible that *V*_*j*_ will be smaller than *V*_*j* − 1_. As a consequence, the path (*Z*_*j*_, *V*_*j*_) of the sequential meta-analysis will go backwards. The situation also has implications for the Christmas tree correction, and if *V*_*j*_ is smaller than *V*_*j* − 1_ we set *H*′_*j*_ = *H*, so that a correction is only made if the path goes forwards. Within a repeated confidence interval framework, a decrease from *V*_*j* − 1_ to *V*_*j*_ yields a confidence interval wider than the previous one. We consider that the possibility that the confidence intervals can become wider (or that the path goes backwards)—and the actual widening of the confidence intervals—is a suitable reflection of the increased uncertainty resulting from identifying inconsistency among the studies.

In Section 3 we consider five methods for addressing the heterogeneity variance in a sequential meta-analysis: (i) ignoring it (a fixed-effect meta-analysis); (ii) implementing a standard random-effects method (using the DerSimonian–Laird estimate); (iii) accounting for uncertainty in τ^2^ (using the approach of Biggerstaff and Tweedie 28); (iv) Bayesian updating of uncertainty in τ^2^; and (v) approximate Bayesian updating of uncertainty in τ^2^. The last two approaches are proposed to allow for prior information on τ^2^ to be incorporated in the early stages of the sequential meta-analysis. We refer to the sequential approaches using Bayesian inference for τ^2^ as ‘semi-Bayes’ to reflect the fact that inference on the parameter of interest (µ) is frequentist. We compare the five approaches, contrasting them with a naïve cumulative random-effects meta-analysis without correction for multiple looks, in a simulation study in Section 4.

## 3. Specific methods for sequential meta-analysis

### 3.1. Fixed-effect sequential meta-analysis using the general parametric approach

For a fixed-effect sequential meta-analysis, as described by Whitehead 9, we take 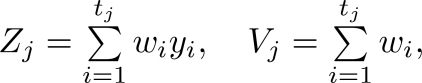 where *y*_*i*_ and *w*_*i*_ are as defined in Section 2.1. As with all methods described below, we apply the confidence interval formulae in Equation (1) to produce repeated confidence intervals.

### 3.2. Random-effects sequential meta-analysis using the general parametric approach

For a random-effects sequential meta-analysis, also described by Whitehead, we take 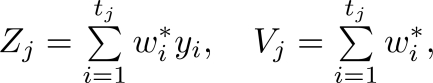 where *y*_*i*_ and 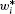 are as defined in Section 2.1 and 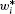 involves the DerSimonian–Laird estimator, 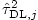, applied to the studies accumulated so far in the sequential meta-analysis. The estimate of 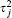 is assumed known at each update, as in a conventional meta-analysis.

### 3.3. Accounting for uncertainty in the estimation of τ^2^

Methods that incorporate uncertainty in 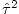 are available (Biggerstaff and Tweedie 28, Hardy and Thompson 29). Biggerstaff and Tweedie consider an approach for accounting for uncertainty in the DerSimonian–Laird estimate based on a gamma approximation to the distribution of *Q*. This leads the authors to a revised weighting scheme for combining the effect estimates, *y*_*i*_: 
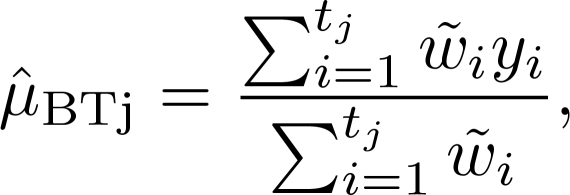
 where 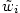 are obtained as the expected values of the DerSimonion–Laird weights, 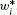, over the approximating distribution for 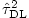 (allowing for truncation at zero), obtained for example using numerical integration.

As the new weights, 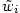, are no longer inverse variances, we define *Z*_*j*_ and *V*_*j*_ as 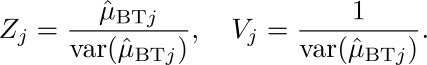 Incorporating uncertainty in estimates of τ^2^ into the meta-analysis should slow the progress of the (*Z, V*) path towards the boundary, since the uncertainty is reflected in a smaller value for *V*. Early repeated confidence intervals are likely to be wide.

### 3.4. Incorporating prior information about heterogeneity: Bayesian updating of τ^2^

A natural approach to making inferences about τ^2^ in the presence of a small number of studies is to draw on external evidence about its likely value. An informative prior distribution may contribute heavily to producing a realistic estimate in the early stages of a sequential meta-analysis. We may therefore expect the estimates of τ^2^ in the meta-analysis to be less erratic, and the path to proceed forward towards the boundaries. We propose to use Bayes' theorem to update the value of τ^2^, and to insert this into the frequentist sequential approach outlined above.

A suitable prior distribution may be developed following the ideas of Higgins and Whitehead 30, who gathered a collection of meta-analyses in the same therapeutic area and produced an empirical distribution from the observed degrees of heterogeneity for a similar type of outcome. A point summary from the prior distribution may be used in the analysis of the first study in the sequential meta-analysis, and point summaries from updated posterior distribution for τ^2^ may be used in subsequent updates of the sequential meta-analysis.

Let *p*(τ^2^) represent the prior distribution for τ^2^, which here will be considered to be an inverse gamma distribution with shape parameter η and scale parameter λ. At the *j*th meta-analysis the likelihood function for the data *L*(µ, τ^2^;{*y*_1_, …, *y*_*t*_*j*__}, {*v*_1_, …, *v*_*t*_*j*__}) is a product of normal distributions with means equal to µand variances equal to (*v*_*i*_ + τ^2^). The joint posterior distribution of µand τ^2^ at this meta-analysis, assuming an independent prior distribution, *p*(µ), for µ, is given by 
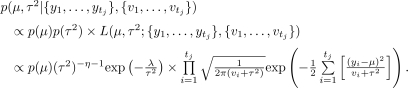
 Inference on τ^2^ would normally be performed by integration or simulation. However, µ is unknown and in our frequentist analysis is not associated with a probability distribution, so cannot be integrated out. A solution is to replace µ by its estimate from the (*j* − 1)th meta-analysis. This makes τ^2^ the only unknown parameter in the posterior formula, and a simple one-dimensional numerical integration can be performed to obtain its posterior mean, 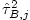 as follows. 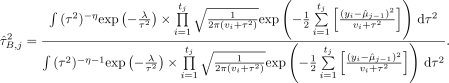 If the first meta-analysis (*j* = 1) contains several studies (i.e. *t*_1_>1), then a suitable value for 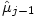 might be provided by the estimate from a standard random-effects meta-analysis based on the first (*t*_1_ − 1) studies. If the first meta-analysis contains exactly two studies (*t*_1_ = 2) we would set 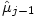 equal to *y*_1_.

We opt for the mean rather than the median or mode since not only is it straightforward to calculate, but it will tend to overestimate τ^2^ in the early stages, producing an analysis that is less likely to yield a false positive result. As information accumulates, and the posterior distribution of τ^2^ develops a stronger peak, the mean will move towards the median and the mode.

For a random-effects sequential meta-analysis, we take 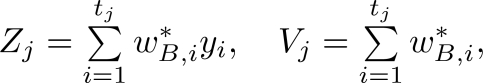 where 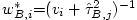. In principle, the approach could be extended to incorporate additional uncertainty in τ^2^ by applying the ideas of Biggerstaff and Tweedie. Specifically, the integrations in the calculation of 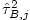 could be performed for the weights 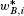 rather than for 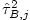. We have not implemented this extension.

### 3.5. An approximation to the Bayesian approach for updating τ^2^

We now propose a simpler approach that avoids the numerical integration. Suppose the true effects in the studies to date were known exactly, so that *y*_*i*_ = θ_*i*_ and *v*_*i*_ = 0. Then the inverse gamma prior distribution for τ^2^ is the conjugate prior distribution for the unknown variance of a normal distribution, and the posterior distribution for τ^2^ is then an inverse gamma posterior distribution with parameters (η + (*t*_*j*_/2)) and 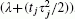, where 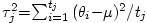 is the heterogeneity variance among the studies up to *t*_*j*_. We may estimate this variance using the DerSimonian–Laird estimate 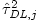. As the mean of an inverse gamma distribution with parameters η and λ is given by λ/(η − 1), it follows that the posterior mean for τ^2^ is given by 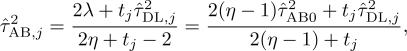 where 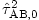 is the mean of the prior inverse gamma distribution. The posterior mean can therefore be viewed as a weighted average of the prior estimate of τ^2^ and the usual estimate from the studies in the meta-analysis. As the number of studies increases, the effect of the prior information decreases.

For a random-effects sequential meta-analysis, our approximate semi-Bayes approach takes 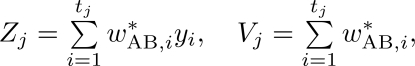 where 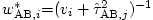. R code for this method is included in the Appendix.

In practice, the approximations here might be expected to yield adequate results when the studies are large (in which case *y*_*i*_≈θ_*i*_ and *v*_*i*_≈0), and to overestimate τ^2^ when the studies are small, due to the overdispersion in the distribution of the estimates, *y*_*i*_, compared with the distribution of the true effects, θ_*i*_.

## 4. Simulation study

### 4.1. Methods of the simulation study

We conducted a simulation study to examine the behaviour of the five sequential meta-analysis methods described in Section 3 under a wide range of conditions. These were also compared with a traditional cumulative meta-analysis approach that simply performs a conventional random-effects meta-analysis on the addition of each study. We chose an O'Brien and Fleming design with 90 per cent power to detect an effect µ_*R*_ of 0.5 with a two-sided significance level of 5 per cent. This value for µ_*R*_ corresponds to a moderately sized standardized mean difference 31, or to an odds ratio, hazard ratio or risk ratio of 1.65. The boundaries for the design, obtained from the package PEST 4.0, are given by *H* = 14.92 and *V*_max_ = 44.32 (see Table I).

The simulation study was conducted with true effect sizes of µ = 0, 0.25, 0.5, and 1, that is ranging from no effect to an effect substantially larger than µ_*R*_. Values for the heterogeneity variance τ^2^ were 0, 0.0625, and 0.25. We simulated studies such that the expected number of studies under the null hypothesis under a fixed-effect analysis would be *t* = 5, *t* = 10, and *t* = 20, as described below. This corresponds to an approximate doubling of sample size for each case we investigated. In total we examined 36 scenarios.

Within each simulated sequential meta-analysis the true effect size from each study, θ_*i*_, was sampled from a *N*(µ, τ^2^) distribution. The mean within-study variance of the effect estimates, *y*_*i*_, across all studies was taken to be σ^2^ = *t*/*V*_max_, ensuring that there would be approximately *t* studies in a sequential fixed-effect meta-analysis if the vertical boundary was crossed at *V*_max_ and τ^2^ = 0. The within-study variance of the effect estimate for each study was sampled from a uniform distribution in the range (σ^2^ − 0.75σ^2^, σ^2^ + 0.75σ^2^). This ensures that the studies have different degrees of precisions, reflecting a realistically wide range of study sizes within each meta-analysis. As an approximate interpretation, for the case of one-group studies estimating means with a between-person standard deviation equal to 4 in every study, this ensures that, for *t* = 5, sample sizes would be distributed between 81 and 567 rather than all being equal to 142. The prior distribution for τ^2^ for the semi-Bayes methods was an inverse gamma distribution with parameters η = 1.5 and λ = 0.08. This corresponds to a prior estimate for τ^2^ of 0.16. This is less than the maximum simulated heterogeneity, and, if the true effect size were 0.5, allows for some studies to have negative effect sizes. Taking η = 1.5 is equivalent to having prior information with the weight of one study, so the prior estimate contributes less than 10 per cent towards the posterior estimate of heterogeneity after ten studies have been included in the meta-analysis.

For each of the 36 scenarios 5000 sequential meta-analyses were simulated and analysed using each of the six approaches, using the procedures described in Section 2. The stopping rule for the naive cumulative meta-analysis method was to stop (conclude an effect exists) if the naive 95 per cent confidence interval excludes 0; or to stop (conclude no effect) if *V*_*j*_ exceeds *V*_max_; or otherwise to continue. The simulation was conducted in *R* (the program is available upon request).

From the simulations, for each approach, we recorded (i) the mean number of studies taken to reach a stopping boundary of the design; (ii) the proportion of simulations stopping having crossed the upper boundary (in order to evaluate Type I error under the null hypothesis); (iii) the percentage of simulations in which all calculated two-sided 95 per cent confidence intervals contained the true effect size µ (that is, the coverage of all confidence intervals); (iv) the percentage of simulations in which the confidence interval at the time when a stopping boundary is crossed (based on *Z*_end_ and *V*_end_) contained the true effect size µ(coverage of the last confidence interval); (v) estimates of the heterogeneity variance, τ^2^, at the time when a stopping boundary is crossed and (vi) mean value of *V*_end_.

### 4.2. Results of the simulation study

Table II–IV give a selection of the results when *t* = 5 (few, large studies), *t* = 10 (more, average sized studies) and *t* = 20 (many, small studies). The results of the simulations should be interpreted in comparison with the fixed-effect sequential method as well as in isolation. The fixed-effect sequential method should in theory have good properties in the absence of heterogeneity. However, the results do not agree with the expectations due to a combination of (i) the inadequacy of the Christmas tree correction; (ii) our random sampling of within-study variances from a uniform distribution, which means that we cannot guarantee to hit the *V*_max_ boundary with the *t*th study and (iii) the *ad hoc* decision to consider an effect present when *V*_end_>*V*_max_ and |*Z*_end_|≥*H*′_end_, a region in which sequential theory does not formally apply. The third issue is expected to increase the probability of declaring that an effect exists, with a larger increase the further away *V*_end_ is from *V*_max_. When µ = 0, the vertical boundary will be crossed in the majority of simulations, and it can be seen from Table II (τ^2^ = 0) with approximately five studies to reach *V*_max_ that the Type I error rate is 0.039 instead of 0.025. When there are more and smaller studies, for example in Table IV, *V*_end_ moves closer to *V*_max_ and it can be seen that the Type I error rate reduces to 0.028. In addition, the probability that all confidence intervals up to and including the one at *V*_end_ will contain the true effect size will only be equal to 0.95 if *V*_end_ = *V*_max_. If *V*_end_<*V*_max_, this probability will be greater than 0.95, and if *V*_end_>*V*_max_ this probability will be less than 0.95. The closer that *V*_end_ is to *V*_max_, the closer will be the probability to 0.95. When µ = 0 and τ^2^ = 0, this probability is equal to 0.922 (Table II) and 0.939 (Table IV). The results for the other sequential meta-analysis approaches should be viewed in this light.

**Table II:** Results from simulation study with approximately five studies

µ	τ^2^		Naïve random-effects	Fixed-effect	Random-effects	BT	Approx. semi-Bayes	Semi-Bayes
0	0	Studies at stopping	6.1	5.5	6.4	6.4	7.5	8.6
		*P*(benefit|µ)	0.048	0.039	0.034	0.033	0.017	0.007
		Coverage, all CIs	90.2	92.2	93.1	93.2	96.5	98.4
		Coverage, last CI	90.2	92.2	93.1	93.2	96.5	98.4
		τ^2^ median	0.014	0	0.015	0.015	0.035	0.060
		τ^2^ mean	0	0	0	0	0.021	0.057
		*V* at stopping	48.6	48.7	49.0	49.0	48.5	47.4
	0.0625	Studies at stopping	7.1	5.3	7.7	7.8	8.8	9.2
		*P*(benefit|µ)	0.102	0.095	0.067	0.071	0.045	0.027
		Coverage, all CIs	79.9	81.5	86.2	86.3	91.2	94.6
		Coverage, last CI	79.9	81.5	86.2	86.3	91.2	94.6
		τ^2^ median	0.040	0	0.044	0.044	0.063	0.074
		τ^2^ mean	0.0072	0	0.015	0.14	0.041	0.066
		*V* at stopping	47.7	48.5	48.3	48.3	48.0	47.2
	0.25	Studies at stopping	10.8	4.8	13.1	13.1	14.3	12.7
		*P*(benefit|µ)	0.181	0.225	0.115	0.114	0.084	0.083
		Coverage, all CIs	64.0	55.6	77.7	77.7	83.7	83.9
		Coverage, last CI	64.0	55.6	77.7	77.7	83.7	83.9
		τ^2^ median	0.15	0	0.16	0.17	0.19	0.16
		τ^2^ mean	0.12	0	0.15	0.15	0.18	0.12
		*V* at stopping	45.5	46.3	46.1	46.1	46.2	46.2
0.5	0	Studies at stopping	3.3	3.7	4.3	4.3	5.2	5.9
		*P*(benefit|µ)	0.958	0.948	0.958	0.959	0.968	0.974
		Coverage, all CIs	89.3	91.6	92.5	92.5	95.8	98.2
		Coverage, last CI	96.0	96.3	97.0	96.9	98.4	99.2
		τ^2^ median	0.03	0	0.018	0.010	0.045	0.068
		τ^2^ mean	0	0	0	0	0.032	0.065
		*V* at stopping	22.2	32.0	32.0	32.0	31.1	30.3
	0.0625	Studies at stopping	3.8	3.7	5.2	5.2	6.0	6.2
		*P*(benefit|µ)	0.942	0.909	0.938	0.939	0.947	0.948
		Coverage, all CIs	80.8	80.9	86.7	86.7	91.3	94.6
		Coverage, last CI	92.9	91.5	94.5	94.5	96.7	97.9
		τ^2^ median	0.062	0	0.046	0.046	0.012	0.073
		τ^2^ mean	0.0014	0	0.0046	0.0046	0.043	0.081
		*V* at stopping	21.0	31.4	30.7	30.7	30.6	29.8
	0.25	Studies at stopping	5.3	3.7	8.3	8.3	9.1	8.0
		*P*(benefit|µ)	0.924	0.840	0.909	0.909	0.908	0.901
		Coverage, all CIs	63.8	56.4	77.9	78.1	83.1	83.5
		Coverage, last CI	86.7	79.5	91.8	91.9	94.9	94.6
		τ^2^ median	0.17	0	0.15	0.15	0.15	0.14
		τ^2^ mean	0.10	0	0.11	0.11	0.18	0.11
		*V* at stopping	18.1	30.6	28.4	28.4	28.5	28.2

BT, Biggerstaff and Tweedie.

**Table III:** Results from simulation study with approximately 10 studies

µ	τ^2^		Naïve random-effects	Fixed-effect	Random-effects	BT	Approx. semi-Bayes	Semi-Bayes
0	0	Studies at stopping	10.9	10.5	11.9	11.9	12.5	13.9
		*P*(benefit|µ)	0.075	0.032	0.028	0.027	0.023	0.015
		Coverage, all CIs	84.7	93.2	93.9	93.9	95.1	96.9
		Coverage, last CI	84.7	93.2	93.9	93.9	95.1	96.9
		τ^2^ median	0.022	0	0.025	0.022	0.033	0.067
		τ^2^ mean	0	0	0	0	0.015	0.065
		*V* at stopping	46.3	46.4	46.6	46.6	46.7	46.1
	0.0625	Studies at stopping	11.6	10.3	13.4	13.4	14.0	14.5
		*P*(benefit|µ)	0.108	0.057	0.046	0.046	0.037	0.027
		Coverage, all CIs	77.4	88.5	90.9	90.9	92.5	94.5
		Coverage, last CI	77.4	88.5	90.9	90.9	92.5	94.5
		τ^2^ median	0.049	0	0.057	0.057	0.067	0.079
		τ^2^ mean	0.0066	0	0.024	0.024	0.038	0.071
		*V* at stopping	46.0	46.4	46.5	46.5	46.5	46.0
	0.25	Studies at stopping	15.1	9.7	19.5	19.5	19.9	17.5
		*P*(benefit|µ)	0.177	0.136	0.069	0.070	0.060	0.063
		Coverage, all CIs	64.9	71.8	85.9	85.8	87.8	87.7
		Coverage, last CI	64.9	71.8	85.9	85.8	87.8	87.7
		τ^2^ median	0.16	0	0.19	0.19	0.20	0.15
		τ^2^ mean	0.13	0	0.18	0.18	0.19	0.12
		*V* at stopping	45.1	45.9	45.7	45.8	45.8	45.7
0.5	0	Studies at stopping	5.1	7.0	7.9	7.9	8.5	9.4
		*P*(benefit|µ)	0.958	0.933	0.943	0.944	0.942	0.947
		Coverage, all CIs	85.9	93.6	94.6	94.6	95.5	97.4
		Coverage, last CI	96.3	97.7	98.1	98.1	98.2	99.0
		τ^2^ median	0.043	0	0.031	0.031	0.048	0.073
		τ^2^ mean	0	0	0	0	0.023	0.069
		*V* at stopping	16.1	30.0	29.7	29.7	29.6	29.5
	0.0625	Studies at stopping	5.6	7.0	9.0	9.0	9.5	9.7
		*P*(benefit|µ)	0.945	0.903	0.922	0.922	0.926	0.932
		Coverage, all CIs	78.2	87.9	90.8	90.9	92.7	94.6
		Coverage, last CI	94.3	95.7	96.8	96.8	97.4	98.1
		τ^2^ median	0.075	0	0.061	0.061	0.077	0.085
		τ^2^ mean	0	0	0.013	0.014	0.037	0.076
		*V* at stopping	15.4	29.9	29.7	29.7	29.7	29.4
	0.25	Studies at stopping	7.1	6.8	12.3	12.4	12.7	11.3
		*P*(benefit|µ)	0.938	0.861	0.909	0.909	0.909	0.901
		Coverage, all CIs	65.0	72.1	85.4	85.2	87.0	86.5
		Coverage, last CI	89.9	90.2	95.5	95.5	95.9	95.5
		τ^2^ median	0.18	0	0.18	0.18	0.19	0.14
		τ^2^ mean	0.11	0	0.15	0.15	0.18	0.11
		*V* at stopping	13.5	29.1	28.1	28.1	28.3	28.4

BT, Biggerstaff and Tweedie.

**Table IV:** Results from simulation study with approximately 20 studies

µ	τ^2^		Naïve random-effects	Fixed-effect	Random-effects	BT	Approx. semi-Bayes	Semi-Bayes
0	0	Studies at stopping	19.7	16.3	22.5	22.5	22.8	24.2
		*P*(benefit|µ)	0.097	0.028	0.023	0.023	0.021	0.016
		Coverage, all CIs	80.7	93.9	94.7	94.8	95.2	96.3
		Coverage, last CI	80.7	93.9	94.7	94.8	95.2	96.3
		τ^2^ median	0.035	0	0.038	0.038	0.044	0.073
		τ^2^ mean	0	0	0	0	0.0008	0.069
		*V* at stopping	45.3	45.4	45.5	45.5	45.5	45.3
	0.0625	Studies at stopping	20.3	20.3	24.1	24.1	24.4	24.8
		*P*(benefit|µ)	0.122	0.040	0.036	0.032	0.031	0.025
		Coverage, all CIs	76.1	91.5	93.1	93.2	93.6	95.0
		Coverage, last CI	76.1	91.5	93.1	93.2	93.6	95.0
		τ^2^ median	0.063	0	0.071	0.071	0.076	0.084
		τ^2^ mean	0.0017	0	0.023	0.023	0.031	0.076
		*V* at stopping	45.2	45.4	45.5	45.5	45.5	45.2
	0.25	Studies at stopping	23.7	19.7	30.6	30.6	30.7	27.2
		*P*(benefit|µ)	0.157	0.079	0.046	0.045	0.044	0.049
		Coverage, all CIs	67.0	83.7	90.5	90.5	90.8	89.8
		Coverage, last CI	67.0	83.7	90.5	90.5	90.8	89.8
		τ^2^ median	0.17	0	0.21	0.21	0.21	0.14
		τ^2^ mean	0.14	0	0.20	0.20	0.20	0.11
		*V* at stopping	44.8	45.2	45.3	45.3	45.3	45.2
0.5	0	Studies at stopping	8.6	13.6	15.2	15.2	15.5	16.3
		*P*(benefit|µ)	0.952	0.910	0.918	0.918	0.920	0.929
		Coverage, all CIs	79.9	93.4	94.4	94.4	94.7	95.8
		Coverage, last CI	95.8	97.8	97.8	97.8	97.9	98.7
		τ^2^ median	0.067	0	0.049	0.049	0.058	0.081
		τ^2^ mean	0	0	0	0	0.016	0.076
		*V* at stopping	13.6	29.3	29.1	29.1	29.1	28.9
	0.0625	Studies at stopping	9.0	13.6	16.2	16.2	16.4	16.6
		*P*(benefit|µ)	0.952	0.897	0.913	0.913	0.914	0.919
		Coverage, all CIs	75.8	91.7	93.3	93.4	93.9	95.4
		Coverage, last CI	95.0	97.6	98.1	98.1	98.0	98.6
		τ^2^ median	0.092	0	0.079	0.079	0.087	0.090
		τ^2^ mean	0	0	0.015	0.015	0.028	0.081
		*V* at stopping	13.1	29.2	28.8	28.8	28.8	29.0
	0.25	Studies at stopping	10.1	13.2	19.8	19.9	20.0	17.8
		*P*(benefit|µ)	0.950	0.878	0.908	0.908	0.907	0.896
		Coverage, all CIs	66.0	84.0	90.8	90.8	90.7	89.6
		Coverage, last CI	91.7	94.8	97.1	97.1	97.1	96.8
		τ^2^ median	0.20	0	0.20	0.20	0.21	0.11
		τ^2^ mean	0.10	0	0.17	0.17	0.18	0.14
		*V* at stopping	11.3	28.0	27.8	27.8	27.9	28.1

BT, Biggerstaff and Tweedie.

The Biggerstaff and Tweedie method is not noticeably different from the sequential random-effects meta-analysis. This is because it does not change the estimate of heterogeneity, but widens the confidence interval in line with the uncertainty in the estimation of heterogeneity. This difference in confidence intervals appears to be small after the first few studies, so the Biggerstaff and Tweedie adjustment has little impact on the analysis.

The simulation results show that the sequential random-effects meta-analysis generally takes at least one study more to reach a stopping boundary than the sequential fixed-effect meta-analysis, the additional number of studies increasing with increasing heterogeneity. The semi-Bayes methods generally result in about one more study than the sequential random-effects meta-analysis. This shows that taking account of heterogeneity can reduce the risk of early stopping.

As the number of studies increases it can be seen that the Type I error rate for the naïve cumulative random-effects meta-analysis without correction for multiple looks also increases, in contrast to the other random-effects approaches which allow for the multiple looks. Type I error rates increase with increasing heterogeneity, this being most noticeable for the sequential fixed-effect meta-analysis, which makes no allowance for heterogeneity. The use of a sequential random-effects meta-analysis improves the Type I error rate in the presence of heterogeneity, but it is still unacceptably high when there is a moderate amount of heterogeneity. Hence these methods may produce spurious findings if there is heterogeneity. The semi-Bayes methods produce values closer to the desired value, but they too show a slight increase with heterogeneity.

The semi-Bayes methods show considerably greater coverage than the sequential fixed-effect meta-analysis when there is no heterogeneity. This over-coverage is caused by their invalid prior assumption of non-zero heterogeneity. The coverage falls off rapidly with heterogeneity for the sequential fixed and random-effects meta-analyses. This reduction in coverage is smaller for the semi-Bayes methods.

There is some underestimation of heterogeneity in the sequential random-effects meta-analysis, which may lead to smaller confidence intervals and hence to early stopping. The semi-Bayes methods provide estimates of τ^2^ that are closer to the true value provided that τ^2^ is not equal to 0. If there is no heterogeneity the Bayesian methods give an overestimate.

In conclusion, the semi-Bayes and approximate semi-Bayes methods had the most desirable false-positive and coverage properties in our simulation study, despite the prior distribution being informative and derived independently of the simulated meta-analyses. Our findings may be dependent on the choice of prior distribution, and we return to this issue in the Discussion.

## 5. Application

**Figure 2:**
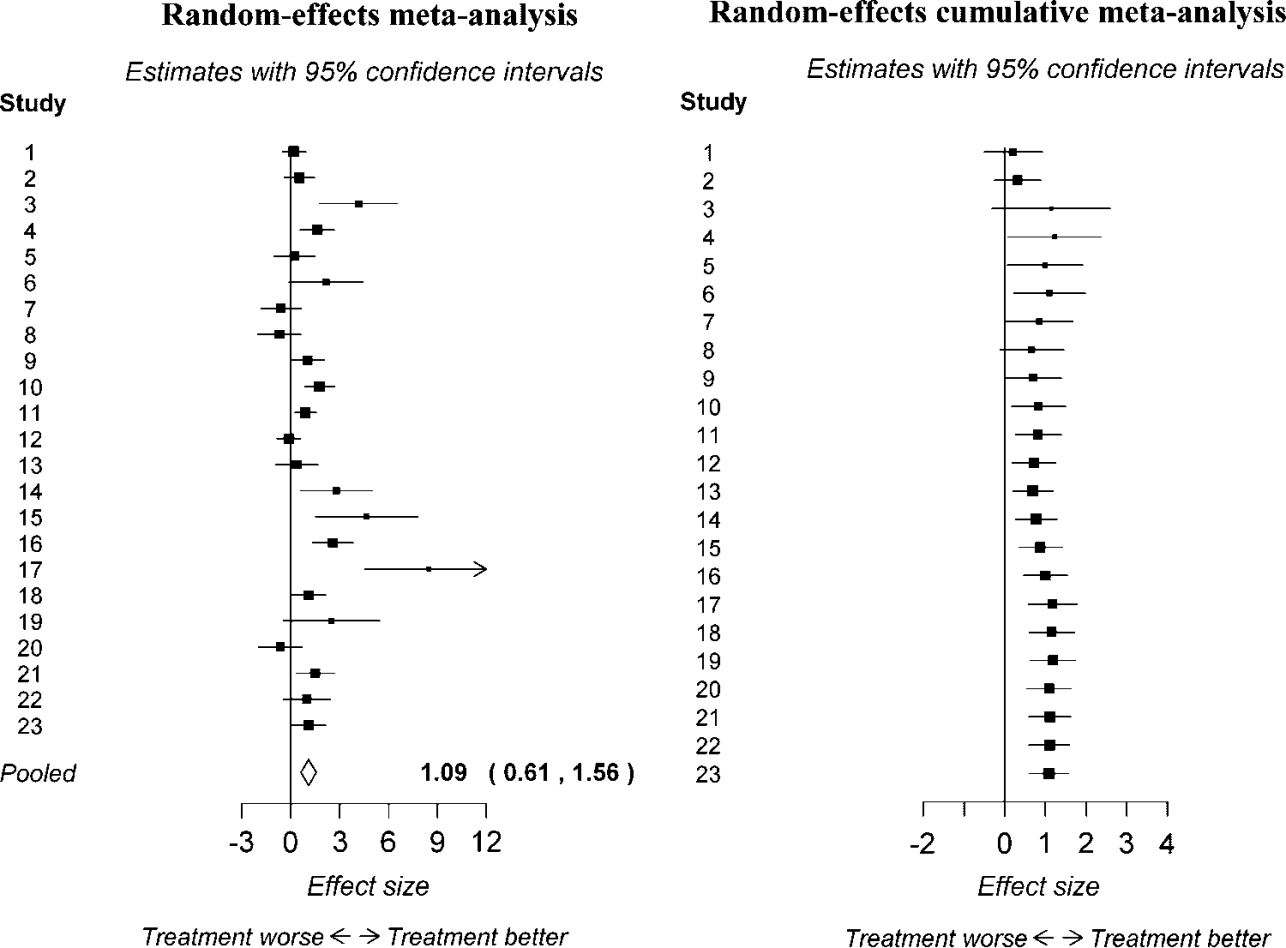
Random-effects meta-analysis and naïve random-effects cumulative meta-analysis of peptic ulcers data.

Sacks *et al.* consider the data from 23 trials that compare endoscopic haemostasis with a control treatment for the treatment of bleeding peptic ulcers 32. The outcome of interest is post-treatment bleeding and the treatment effect recorded here is the log-odds ratio of ‘no bleeding’. The trial estimates *y*_*i*_ and *v*_*i*_ are calculated from Wald statistics, that is 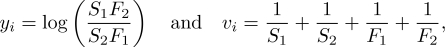 where *S*_1_ and *F*_1_ are the number of patients with ‘no bleeding’ and ‘bleeding’ in the endoscopic haemostasis group, and *S*_2_ and *F*_2_ are defined similarly for the control group. A positive value for *y*_*i*_ favours the experimental treatment. The data and log-odds ratio estimates and confidence intervals for the individual studies are presented in Table V. For trials in which there were no patients with ‘no bleeding’ or no patients with ‘bleeding’ in at least one treatment group, 0.5 was added to all of the four cells of the 2 × 2 contingency table. Although this is common practice, for meta-analyses of rare events it is often preferable to use alternative approaches such as logistic regression 33. However, such methods do not fall conveniently into the general sequential framework we describe. A conventional random-effects meta-analysis of the full data set (Figure 2) demonstrates a significant benefit of treatment, with a log-odds ratio of 1.09 (an odds ratio of 3.0), but with considerable heterogeneity 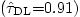.

**Table V:** Peptic ulcers data: randomized trials of endoscopic hemostasis compared with control for bleeding peptic ulcers: Log-odds ratio of ‘no bleeding’

	Hemostasis		Control						
							
Trial, *i*	Bled	Total	Bled	Total	*w*_*i*_	*w*_*i*_*y*_*i*_	Log-odds ratio (*y*_*i*_) [odds ratio]		95 per cent CI 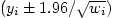
1. Vallon (1980)	20	68	23	68	7.32	1.50	0.20	[1.2]	−0.52, 0.93
2. Swain (1981)	11	36	17	40	4.29	2.22	0.52	[1.7]	−0.43, 1.47
3. Papp (1982)	1	16	13	16	0.68	2.83	4.17	[65]	1.79, 6.56
4. Rutgeerts (1982)	5	52	19	54	3.31	5.39	1.63	[5.1]	0.55, 2.71
5. MacLeod (1983)	6	21	8	24	2.38	0.53	0.22	[1.3]	−1.05, 1.49
6. Jensen (1984)	2	7	7	9	0.74	1.62	2.17	[8.8]	−0.10, 4.44
7. Kernohan (1984)	9	21	7	24	2.52	−1.51	-0.60	[0.5]	−1.83, 0.63
8. Goudie (1984)	7	21	5	25	2.15	−1.49	-0.69	[0.5]	−2.03, 0.64
9. Freitas (1985)	7	36	17	42	3.62	3.75	1.04	[2.8]	0.01, 2.07
10. Swain (1986)	7	69	27	68	4.54	8.00	1.76	[5.8]	0.84, 2.68
11. O'Brien (1986)	17	101	34	103	8.72	7.76	0.89	[2.4]	0.23, 1.55
12. Krejs (1987)	19	85	18	89	7.28	−0.92	−0.13	[0.9]	−0.85, 0.60
13. Brearley (1987)	6	20	8	21	2.27	0.82	0.36	[1.4]	−0.94, 1.66
14. Moreto (1987)	1	16	11	21	0.80	2.23	2.80	[16.5]	0.61, 5.00
15. Laine (1987)	0	10	12	14	0.39	1.81	4.65	[105]	1.51, 7.80
16. Panes (1987)	3	55	25	58	2.36	6.09	2.57	[13.1]	1.30, 3.85
17. Chung (1987)	0	34	34	34	0.25	2.09	8.47	[4761]	4.52, 12.4
18. Balanzo (1988)	7	36	15	36	3.43	3.72	1.08	[3.0]	0.03, 2.14
19. Fellerton (1989)	0	20	5	23	0.44	1.09	2.50	[12]	−0.46, 5.46
20. Angerinas (1989)	7	33	4	32	2.14	−1.36	−0.63	[0.5]	−1.97, 0.71
21. Rutgeerts (1989)	10	40	12	20	2.93	4.40	1.50	[4.5]	0.36, 2.65
22. Chiozzini (1989)	4	34	5	19	1.80	1.78	0.99	[2.7]	−0.47, 2.45
23. Laine (1989)	7	38	15	37	3.48	3.85	1.11	[3.0]	0.05, 2.16

We suppose for the purposes of illustration that this meta-analysis had been planned with the aim of incorporating observations at the end of each trial as they became available. Given the anticipated heterogeneity of effects, we apply the random-effects methods described above. The analysis uses an O'Brien and Fleming design in order to avoid early stopping unless the odds ratio is very large, with a significance level of 5 and 90 per cent power to detect a log-odds ratio of 0.693 (an odds ratio of 2). From Table I, for µ_*R*_ = 0.693 this gives *H* = 10.77 and *V*_max_ = 23.07 (Figure 1).

**Figure 3:**
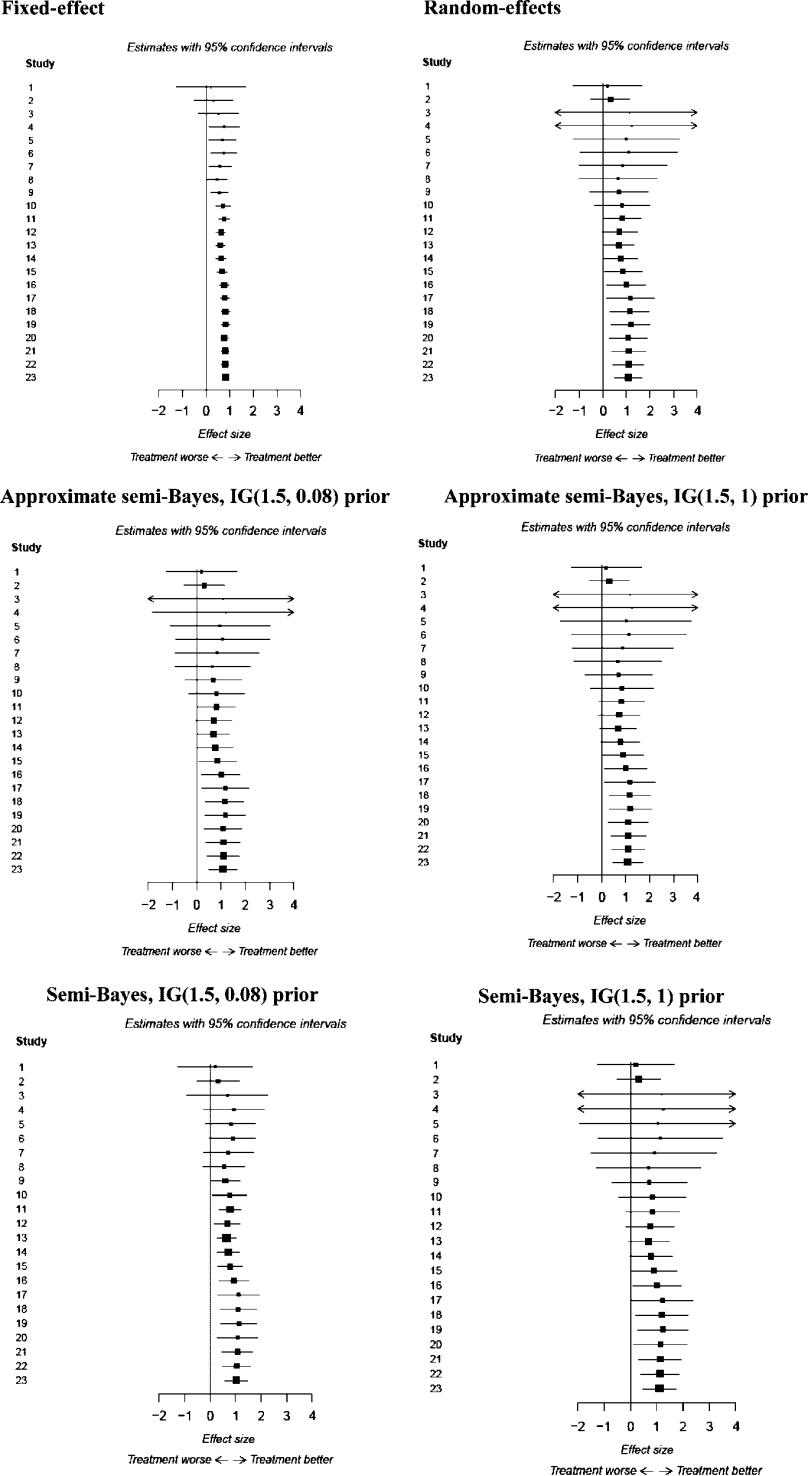
Sequential meta-analysis of peptic ulcers data.

The sequential random-effects meta-analysis and the approximate semi-Bayes and semi-Bayes methods are applied and compared with a sequential fixed-effect meta-analysis (Figure 3). To assess sensitivity to prior distributions, two inverse gamma prior distributions for τ^2^ are used for the semi-Bayes methods: an IG (1.5, 0.08) has a prior mean of 0.16 and an IG (1.5, 1) has a prior mean of 2. These prior means are respectively much smaller and much larger than the DerSimonian–Laird estimate based on all of the trials. Table VI shows the number of studies included at the point when the stopping criterion is met, together with estimates of the treatment effect (*Z*_end_/*V*_end_) and heterogeneity, and the last repeated confidence interval. If the plan for this sequential meta-analysis had been to follow the formal stopping rule of the O'Brien and Fleming design, then it would be more appropriate to present a ‘final’ analysis which correctly adjusts for earlier looks. The results from conducting this ‘final’ analyses using the PEST package 17 are included in Table VI. Such an analysis corrects for the bias in the *Z*_end_/*V*_end_estimate, and provides an appropriate *P* value and confidence interval. The confidence interval is narrower than the last repeated confidence interval, because the repeated confidence intervals have had no effect on the decision to conduct further studies.

**Table VI:** Results from the sequential meta-analyses of the peptic ulcers data

		Fixed	Random	Approx semi-Bayes IG(1.5, 0.08)	Approx semi-Bayes IG(1.5, 1)	Semi-Bayes IG(1.5, 0.08)	Semi-Bayes IG(1.5, 1)
At stopping	Number of trials	4	11	11	15	9	15
	*Z*_end_/*V*_end_	0.77	0.82	0.82	0.89	0.61	0.90
	Last						
	confidence	0.14, 1.39	0.014, 1.63	0.042, 1.59	0.032, 1.75	0.015, 1.20	0.0054, 1.79
	interval						
	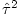	0	0.55	0.52	0.74	0.17	0.79
PEST analysis	*P*-value	0.0055	0.0052	0.0041	0.0048	0.0161	0.0039
	Median	0.74	0.81	0.82	0.81	0.60	0.84
	unbiased estimate of µ [odds ratio] Confidence	[2.1]	[2.2]	[2.3]	[2.2]	[1.8]	[2.3]
	interval for µ	0.22, 1.24	0.24, 1.38	0.26, 1.37	0.25, 1.37	0.11, 1.09	0.27, 1.41

These results show that a sequential fixed-effect meta-analysis, which ignores the heterogeneity, stops after four studies (see also Figure 1), leading to a smaller estimate of the treatment effect compared with that from all trials and a confidence interval that is narrow. The sequential random-effects method requires 11 studies, and produces a slightly larger estimate of the treatment effect and a wider confidence interval. When the prior distribution provides a much smaller underestimate of τ^2^ than is apparent in the dataset, as with the IG (1.5, 0.08) prior, the semi-Bayes method stops after a smaller number of studies (nine) although the approximate semi-Bayes method carries on for the same 11 studies as for the sequential random-effects method. The earlier stopping of the semi-Bayes IG (1.5, 0.08) method results in smaller estimates of treatment effect and heterogeneity. The use of an IG (1.5, 1) prior with its large prior estimate of heterogeneity causes the approximate semi-Bayes and semi-Bayes methods to stop after 15 trials. The estimates of treatment effect and heterogeneity from the sequential approaches are smaller than those from the overall random effects analysis of 23 studies. This is mainly due to the seventeenth trial, which has a large, beneficial effect of treatment, increasing the heterogeneity.

In our example, we have assumed that a sequential approach to the meta-analysis had been planned before the trials had reported their results. The example is instructive in illustrating some of the limitations of applying our generic statistical methods prospectively in a meta-analysis situation. We have remarked already that 2 × 2 contingency tables may be better analysed using binomial likelihoods. This would have avoided the need to make continuity corrections by adding 0.5 s to cell counts. Furthermore, the use of maximum likelihood estimates of odds ratios also creates a correlation between the *y*_*i*_ and *v*_*i*_. Indeed, a commonly applied test for association between the estimates and standard errors produces *P* = 0.005 34, although a more appropriate test based on efficient score and Fisher information statistics, which overcomes artefactual correlation between the estimates and weights, produces *P* = 0.44 35. Also, some of the studies display extreme treatment effects; for example, in the Chung study, 0 and 100 per cent of the patients bled in the treatment and control groups, respectively. This study is not very informative about the magnitude of the odds ratio, and receives very little weight in the meta-analysis, despite providing convincing evidence that the treatment has a large effect on the absolute risk of bleeding. Using risk differences rather than odds ratios as the effect size would have given this study more weight, but risk differences are generally discouraged as a metric for meta-analysis 36.

## 6. Discussion

This paper has considered the use of sequential methods for meta-analyses that incorporate random effects to allow for heterogeneity between studies. Our emphasis has been on determining a straightforward approach that has good empirical properties. We have developed and compared five methods, including a direct extension of the standard random-effects meta-analysis method, an extension of the approach of Biggerstaff and Tweedie, a semi-Bayes method involving Bayesian updating of the heterogeneity variance, and an approximation to the semi-Bayes method. The choice of an O'Brien and Fleming sequential design with a Christmas tree correction for discrete monitoring leads to a simple procedure for calculating repeated confidence intervals, which enables us to present sequential meta-analyses using forest plots, the conventional way of illustrating meta-analysis results. When either a repeated confidence interval excludes zero or *V*_max_ is reached, then the recommendation can be made that no further research is required to inform the question addressed by the meta-analysis.

Some issues are worthy of discussion, including philosophical, practical, and technical considerations. First, should sequential methods be applied to meta-analysis? Second, if sequential methods are to be used, what methods should be implemented in practice? Third, what technical problems remain that require further research?

The use of sequential methods for meta-analysis is contentious. Whereas the steering committee for a prospective study has control over the recruitment of participants, researchers undertaking meta-analyses may have no direct control over whether further studies are performed. Whether sequential methods have a role for updating of meta-analyses that are unconnected with primary researchers (such as those in Cochrane reviews) remains an open question. Chalmers and Lau 37 question the need to correct for multiple looks within a cumulative meta-analysis because of the lack of direct control. However, we would remark that many systematic reviews contain recommendations for practice and for research, and decisions over the content of these recommendations are similar to decisions over whether a primary research study should continue to recruit participants. Formal sequential methods for this process allow the wider field of evolving knowledge to be subject to the same rigorous considerations of Type I and Type II errors that individual randomized trials have traditionally received.

Meta-analyses are now well established as part of the research process, and many have argued for the need for primary studies to both be informed by meta-analyses of existing evidence, and be rounded off with an updated meta-analysis including the newly generated evidence. Thus primary research is increasingly viewed as part of a wider sequential process, and the methods described here may have application in this context. For instance, there may well be more enthusiasm for conducting a new study if the effect estimate is close to an upper or lower stopping boundary in a scheme such as Figure 1. Finally, we believe that sequential meta-analyses can play an important role in the actual design of individual studies, since the amount of further information that would be required to meet a pre-specified *V*_max_ can be determined. However, there are many considerations in the design of a new study, and it is rare for them to be designed primarily around an updated meta-analysis. Related issues are discussed by Sutton *et al.* 38.

One area of potential application of sequential methods is the monitoring of adverse effects of pharmacological interventions. However, as we remark in Section 5, the particular general parametric approach we describe does not lend itself well to rare events. Furthermore, it is not clear that strict control over false-positive findings is important in this context, since a small, non-statistically significant, signal should still be investigated when the adverse effect is major. More research is required to explore appropriate methods for this issue.

If sequential methods are to be implemented, can the methods we have described be recommended? Certainly we would recommend against a fixed-effect approach. However, in a random-effects framework, a key problem in the early stages of a sequential meta-analysis is the poor estimation of the heterogeneity variance. We argue that prior information is therefore necessary. Of our two semi-Bayes methods, the approximate semi-Bayes approach is straightforward to implement, and this is the one we recommend from among those we investigated. From the simulation results, it appears that the method has reasonable false-positive and coverage properties, although these are likely to be dependent on the choice of prior distribution for the among-study variation. Our application demonstrated that the early stages of the sequential meta-analysis can be affected by the prior distribution. Further empirical research is needed to characterize the degree of heterogeneity that can be anticipated in a meta-analysis with particular clinical and methodological features, so that realistic informative prior distributions can be formulated. An alternative approach would be a sensitivity analysis in which the sequential meta-analysis is repeated for a variety of plausible values of τ^2^, but an explicit stopping rule does not follow from such an approach.

Our recommendations are interim, since more work is required in this area. We have commented on methods based on the law of the iterated logarithm proposed by Lan *et al.* 13, 14. Their open-ended boundaries have the advantage of allowing studies to continue being conducted as long as the null hypothesis is not rejected, but they do not ensure that a pre-specified power is achieved. Open-ended boundaries with specified Type 2 error may offer preferable methods. Some technical issues with the proposed methods might also be investigated. For example, we have assumed that primary inference is to be made on the mean effect across studies. This does not take into account the extent of heterogeneity. It may be more appropriate to base decisions on alternative characteristics of the random-effects distribution, such as the probability that an individual study will have an effect of a particular size. Such inferences can be drawn from the predictive distribution of the effect in a new study 24. Another limitation is that we have proposed the calculation of repeated confidence intervals until and including the first time that *V*_max_ is exceeded. When this last repeated confidence interval is calculated, there is a probability of slightly less than 1 − α that the truth is contained in every interval. The reduction in the probability depends on the increase in *V* from *V*_max_, and will be greater if only a few sequential steps have been conducted. A better understanding of this issue would be useful. Finally, the advantage of using the Christmas tree correction for discrete monitoring with the O'Brien and Fleming design is that this leads to a simple procedure for calculating the repeated confidence intervals, but there are also some disadvantages to this approach. First, Stallard and Facey 39 show that the Christmas tree correction is less accurate for the O'Brien and Fleming design than for other types of sequential design, particularly if there are only a few looks at the data. Second, the Christmas tree correction depends on the information at the current (*V*_*j*_) and previous (*V*_*j* − 1_) meta-analysis update. If (*V*_*j*_ − *V*_*j* − 1_) is less than 0, there is no Christmas tree correction. However, if at the next update (*V*_*j* + 1_ − *V*_*j*_) is greater than 0, there will be a Christmas tree correction. However, *V*_*j* + 1_ may be less than *V*_*j* − 1_. Further work is needed to assess the effect that this might have on the validity of the results.

We have considered a frequentist sequential approach to meta-analysis, proposing semi-Bayes approaches to allow prior information about τ^2^ to be incorporated. The entire meta-analysis could alternatively be undertaken within a fully Bayesian framework. The meta-analyst would then calculate a new credibility interval for every meta-analysis update. The credibility interval, unlike the frequentist repeated confidence interval, is not corrected for multiple looks. The posterior distributions of µ and τ^2^ from the first meta-analysis would become the prior distribution for the second and so on. If τ^2^ increases during the course of the sequential meta-analysis, the credibility interval may become wider, but this causes no problem. Bayesian stopping rules for individual clinical trials have been proposed by various authors (see, for example 40, 41). However, as the Bayesian inference is not affected by the repeated updates, Bayesian monitoring procedures can have very poor frequentist properties, such as inflated Type I errors 42.

## References

[1] The Cochrane Collaboration (2010). Cochrane Database of Systematic Reviews. The Cochrane Library.

[2] Higgins JPT, Green S, Scholten RJPM, Higgins JPT, Green S (2008). Maintaining reviews: updates, amendments and feedback. Cochrane Handbook for Systematic Reviews of Interventions.

[3] Simes RJ (1995). Prospective meta-analysis of cholesterol-lowering studies: the prospective pravastatin pooling project (PPP) and the cholesterol treatment trialists (CTT) collaboration. American Journal of Cardiology.

[4] Baigent C, Keech A, Kearney PM, Blackwell L, Buck G, Pollicino C, Kirby A, Sourjina T, Peto R, Collins R, Simes R (2005). Efficacy and safety of cholesterol-lowering treatment: prospective meta-analysis of data from 90 056 participants in 14 randomised trials of statins. The Lancet.

[5] Writer WD, Stienstra R, Eddleston JM, Gatt SP, Griffin R, Gutsche BB, Joyce TH, Hedlund C, Heeroma K, Selander D (1998). Neonatal outcome and mode of delivery after epidural analgesia for labour with ropivacaine and bupivacaine: a prospective meta-analysis. British Journal of Anaesthesia.

[6] Schunkert H, Gotz A, Braund P, McGinnis R, Tregouet DA, Mangino M, Linsel-Nitschke P, Cambien F, Hengstenberg C, Stark K, Blankenberg S, Tiret L, Ducimetiere P, Keniry A, Ghori MJ, Schreiber S, El Mokhtari NE, Hall AS, Dixon RJ, Goodall AH, Liptau H, Pollard H, Schwarz DF, Hothorn LA, Wichmann HE, Konig IR, Fischer M, Meisinger C, Ouwehand W, Deloukas P, Thompson JR, Erdmann J, Ziegler A, Samani NJ (2008). Repeated replication and a prospective meta-analysis of the association between chromosome 9p21.3 and coronary artery disease. Circulation.

[7] Sims AM, Timms AE, Bruges-Armas J, Burgos-Vargas R, Chou CT, Doan T, Dowling A, Fialho RN, Gergely P, Gladman DD, Inman R, Kauppi M, Kaarela K, Laiho K, Maksymowych W, Pointon JJ, Rahman P, Reveille JD, Tuomilehto J, Vargas-Alarcon G, Wordsworth BP, Xu H, Brown MA (2008). Prospective meta-analysis of interleukin 1 gene complex polymorphisms confirms associations with ankylosing spondylitis. Annals of the Rheumatic Diseases.

[8] Berkey CS, Mosteller F, Lau J, Antman EM (1996). Uncertainty of the time of first significance in random effects cumulative metaanalysis. Controlled Clinical Trials.

[9] Whitehead A (1997). A prospectively planned cumulative meta-analysis applied to a series of concurrent clinical trials. Statistics in Medicine.

[10] Whitehead J (1997). The Design and Analysis of Sequential Clinical Trials.

[11] Pogue JM, Yusuf S (1997). Cumulating evidence from randomized trials: utilizing sequential monitoring boundaries for cumulative meta-analysis. Controlled Clinical Trials.

[12] Devereaux PJ, Beattie WS, Choi PT, Badner NH, Guyatt GH, Villar JC, Cina CS, Leslie K, Jacka MJ, Montori VM, Bhandari M, Avezum A, Cavalcanti AB, Giles JW, Schricker T, Yang H, Jakobsen CJ, Yusuf S (2005). How strong is the evidence for the use of perioperative beta blockers in non-cardiac surgery? Systematic review and meta-analysis of randomised controlled trials. BMJ.

[13] Lan KKG, Hu M, Cappelleri JC (2003). Applying the law of iterated logarithm to cumulative meta-analysis of a continuous endpoint. Statistica Sinica.

[14] Hu M, Cappelleri JC, Lan KKG (2007). Applying the law of iterated logarithm to control type I error in cumulative meta-analysis of binary outcomes. Clinical Trials.

[15] Brok J, Thorlund K, Gluud C, Wetterslev J (2008). Trial sequential analysis reveals insufficient information size and potentially false positive results in many meta-analyses. Journal of Clinical Epidemiology.

[16] Wetterslev J, Thorlund K, Brok J, Gluud C (2008). Trial sequential analysis may establish when firm evidence is reached in cumulative meta-analysis. Journal of Clinical Epidemiology.

[17] MPS Research Unit (2000). PEST 4.

[18] Whitehead A, Whitehead J (1991). A general parametric approach to the meta-analysis of randomised clinical trials. Statistics in Medicine.

[19] Shuster JJ (2010). Empirical vs natural weighting in random effects meta-analysis. Statistics in Medicine.

[20] Laird N, Fitzmaurice G, Ding X (2010). Comments on ‘Empirical vs natural weighting in random effects meta-analysis’. Statistics in Medicine.

[21] Waksman JA (2010). Comments on ‘Empirical vs natural weighting in random effects meta-analysis’. Statistics in Medicine.

[22] Thompson SG, Higgins JPT (2010). Comments on ‘Empirical vs natural weighting in random effects meta-analysis’. Statistics in Medicine.

[23] DerSimonian R, Laird N (1986). Meta-analysis in clinical trials. Controlled Clinical Trials.

[24] Higgins JPT, Thompson SG, Spiegelhalter DJ (2009). A re-evaluation of random-effects meta-analysis. Journal of the Royal Statistical Society Series A.

[25] Bollen CW, Uiterwaal CS, van Vught AJ, van dT I (2006). Sequential meta-analysis of past clinical trials to determine the use of a new trial. Epidemiology.

[26] O'Brien PC, Fleming TR (1979). A multiple testing procedure for clinical trials. Biometrics.

[27] Jennison C, Turnbull BW (1989). Interim analyses: the repeated confidence interval approach. Journal of the Royal Statistical Society, Series B.

[28] Biggerstaff BJ, Tweedie RL (1997). Incorporating variability in estimates of heterogeneity in the random effects model in meta-analysis. Statistics in Medicine.

[29] Hardy RJ, Thompson SG (1996). A likelihood approach to meta-analysis with random effects. Statistics in Medicine.

[30] Higgins JPT, Whitehead A (1996). Borrowing strength from external trials in a meta-analysis. Statistics in Medicine.

[31] Cohen J (1988). Statistical Power Analysis for the Behavioral Sciences.

[32] Sacks HS, Chalmers TC, Blum AL, Berrier J, Pagano D (1990). Endoscopic hemostasis: an effective therapy for bleeding peptic ulcers. Journal of the American Medical Association.

[33] Bradburn MJ, Deeks JJ, Berlin JA, Localio AR (2006). Much ado about nothing: a comparison of the performance of meta-analytical methods with rare events. Statistics in Medicine.

[34] Egger M, Davey Smith G, Schneider M, Minder C (1997). Bias in meta-analysis detected by a simple, graphical test. BMJ.

[35] Harbord RM, Egger M, Sterne JA (2006). A modified test for small-study effects in meta-analyses of controlled trials with binary endpoints. Statistics in Medicine.

[36] Deeks JJ, Altman DG, Egger M, Davey Smith G, Altman DG (2001). Effect measures for meta-analysis of trials with binary outcomes. Systematic Reviews in Health Care: Meta-analysis in Context.

[37] Chalmers TC, Lau J (1993). Meta-analytic stimulus for changes in clinical trials. Statistical Methods in Medical Research.

[38] Sutton AJ, Cooper NJ, Jones DR, Lambert PC, Thompson JR, Abrams KR (2006). Evidence-based sample size calculations based upon updated meta-analysis. Statistics in Medicine.

[39] Stallard N, Facey KM (1996). Comparison of the spending function methods and the christmas tree correction for group sequential trials. Journal of Biopharmaceutical Statistics.

[40] Berry DA (1985). Interim analyses in clinical-trials: classical vs bayesian approaches. Statistics in Medicine.

[41] Freedman LS, Spiegelhalter DJ (1989). Comparison of Bayesian with group sequential methods for monitoring clinical trials. Controlled Clinical Trials.

[42] Jennison C, Turnbull BW (2000). Group Sequential Methods with Applications to Clinical Trials.

